# The effect of nitrogen lone-pair interaction on the conduction in a single-molecule junction with amine-Au bonding

**DOI:** 10.1038/s41598-018-22893-7

**Published:** 2018-03-27

**Authors:** Yoshihiro Sugita, Atsushi Taninaka, Shoji Yoshida, Osamu Takeuchi, Hidemi Shigekawa

**Affiliations:** Faculty of Pure and Applied Sciences, University of Tsukuba, Tsukuba, 305-8571 Japan

## Abstract

We have applied our previously developed three-dimensional dynamic probe method to analyze the conductance in a Au-/1,4-benzenediamine (BDA)/Au single molecule junction. This structure is a typically used example to demonstrate the high performance of the break junction (BJ) method for measuring conductance with small variations, however, details of the interaction of the nitrogen (N) lone-pair in the amine group with a Au electrode, which is considered to have a fundamental role in determining the conductance of the single molecule junction with the amine, have not yet been clarified and still remain an important issue to be resolved. In this study, we have succeeded, for the first time, in observing the site-dependent change in conductance of this system while the molecular conformation was accurately controlled, and the results were well reproduced by a simulation taking account of the effect of the N lone-pair in an amine bonding with a Au electrode.

## Introduction

Owing to the variety of functions created by the high designability of molecules, the development of molecular devices has been one of the most attractive targets in recent decades for realizing nanoscale transistors, memories and switching controlled by electric-field, optical and mechanical processes^1–4^. The understanding and control of metal/single molecule/metal junctions is the key factor for achieving such purposes^5,6^. Mechanically controllable break junction (MC-BJ) and scanning tunneling microscope break junction (STM-BJ) methods have been widely applied to analyze the characteristics of single-molecule junctions and many valuable results have been obtained^1,7–18^.

In the STM-BJ method, after forming a Au (electrode)/molecule/Au (electrode) junction for a target molecule, the distance between the electrodes *z* is increased while its conductance *G* is measured. Then the plateau of the *G*-*z* curve upon the breaking of the junction is considered to provide the conductance of the single molecule that remains between the electrodes at the breaking point. Since we generally measure the conductance upon the breaking of junctions, as the name indicates, measurement can be carried out only once for each molecule. Therefore, *G*-*z* curves are measured for many molecules and their values are summarized in a histogram. The variation in the conductance histogram is generally large and its mode is adopted as the single-molecular conductance of the target molecule. Another technique is to move the STM tip back and force without breaking junction to analyze the change in *G* due to the conformational change in junction^10,11^, and site-dependent electric coupling and strain, for example, were clearly observed. However, since bonding sites cannot be determined experimentally, additional ingenuity is desired for further understanding.

To reduce the variation in the observed values, an amine (NH_2_)-Au link has been adopted instead of a thiol-Au link to form a more reproducible molecular junction^8,9^. Namely, the amine link binds preferentially to undercoordinated Au atoms in the junction, and its weaker bond is assumed to induce less change in the Au structure upon the breaking of the molecular junction^19^. In fact, sharper and more reproducible histograms have been obtained by BJs for molecules with amine-Au links^8,9,13–18^. In the case of a BJ of a 1,4-benzenediamine (BDA) single molecule, one of the simplest molecules that can form a single-molecule junction, the variation of its conduction histogram was, for example, only one order around its peak at 0.0064 *G*_0_ (*G*_0_ = 2*e*^2^/*h*, *h* is Planck’s constant)^8,9^. The interaction of the nitrogen (N) lone-pair in the amine group with the Au electrode is considered to play a fundamental role in determining the bonding sites, which, however, has not yet been clarified due to random structures of the Au atoms below the amine-Au bond^9^, and still remains an important issue to be resolved.

To obtain a more detailed understanding of the single-molecule conductance and to develop the functions of single-molecule junctions in a more accurate form for further advances in applications, site-dependent analysis is a key factor. Recently, we have developed a three-dimensional (3D) dynamic probe method^20,21^, based on the point contact method^22^, which enables changes in conductance to be measured, while the adsorption site and molecular angle related to the substrate surface being determined experimentally. In this study, we have applied this method to the analysis of a Au/BDA molecule/Au junction. The changes in *G* were successfully observed while the molecular conformation was accurately controlled and the results were well reproduced by a simulation taking account of the role of the N lone-pair in an amine bonding with a Au electrode.

## Results and Discussion

### Experimental scheme and measurements

**Figure 1:**
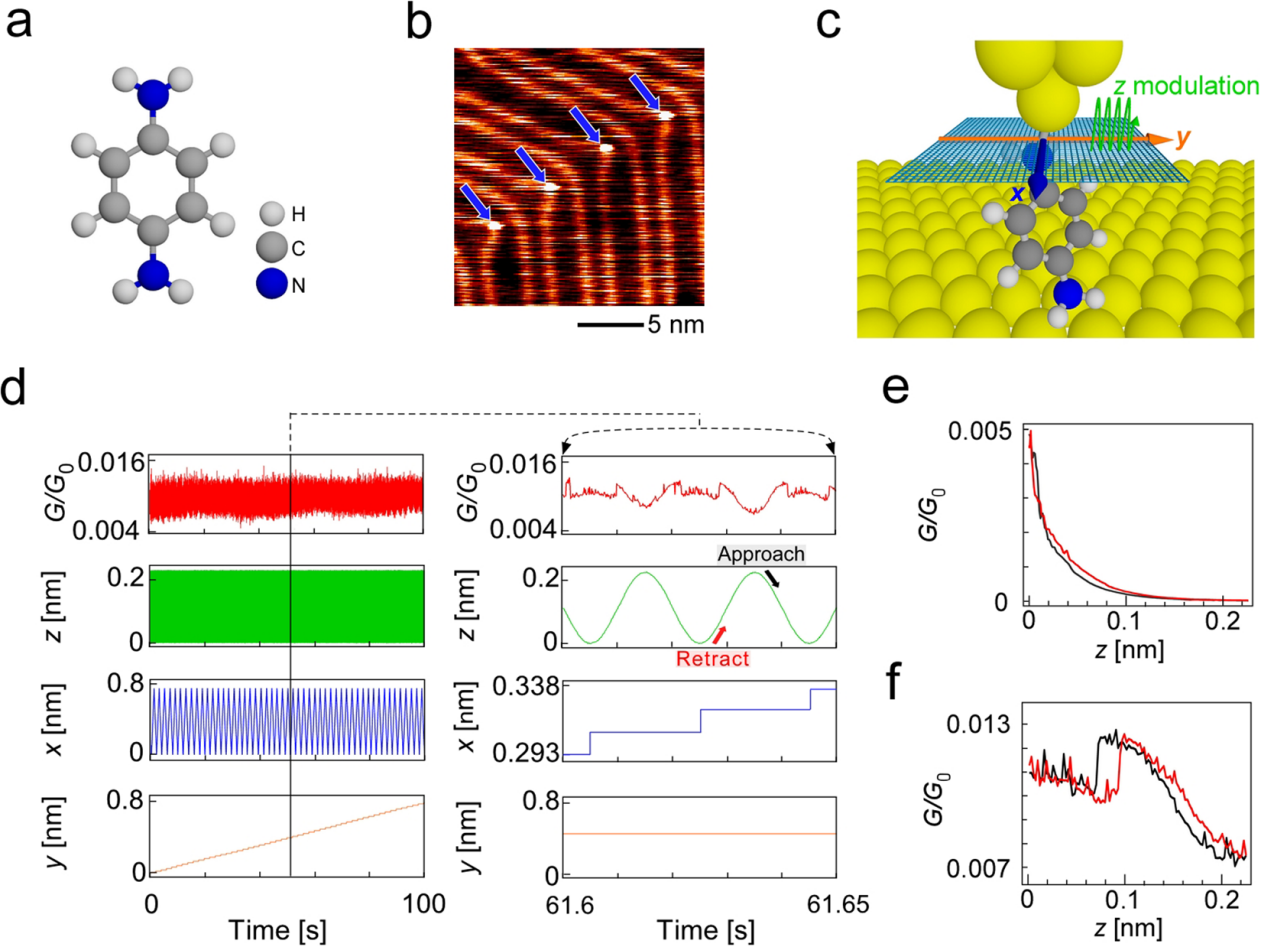
Measurement scheme. (**a**) Schematic structure of BDA molecule. (**b**) STM image of a Au(111) herringbone structure. Blue arrows indicate four deposited BDA molecules. (**c**) Schematic illustration of the 3D dynamic probe method. (**d)** Measurement scheme. Green, blue and orange lines indicate the *z* modulation and *x*- and *y*-scans, respectively and the red line shows the corresponding change in the normalized conductance *G*/*G*_0_ (*G*_0_ = 2*e*^2^/*h*, *h* is Planck’s constant). (**e**,**f**) *G*-*z* curves obtained without and with BDA molecule, respectively. *G*/*G*_0_ indicates the normalized conductance (*G*_0_ = 2*e*^2^/*h*, *h* is Planck’s constant).

Figure 1a shows a schematic illustration of the BDA molecule. Figure 1b is an STM image of a Au(111) surface with four BDA molecules selectively adsorbed on the elbow sites of a herringbone structure like 1,4-benzenedithiol (BDT) and 4,4′-bipyridine (BPY)^21^, as indicated by blue arrows. After observing a target BDA molecule, the STM tip was moved to above it. Then the feedback was turned off and the sample bias voltage was set to 10 mV. The STM tip was moved back and forth in the *z*-direction in accordance with a sinusoidal function and was made slowly approach the target molecule. The formation of a junction was confirmed by a rapid increase in current.

After formation was confirmed, the STM tip was moved back to raise the molecule to have some angle from the substrate for a stable measurement and a two-dimensional scan was carried out step by step after each cycle of *z*-modulation as shown in Fig. 1c. In this case, the angle of the molecule at the start point for measurement was well determined from the analysis of the three-dimensional (3D) conductance map, which will be explained later. Figure 1d shows the measurement scheme, an example of the obtained data and their magnifications. The green, blue, and orange lines indicate the *z* modulation and *x*- and *y*-scans, respectively (see Methods for details) and the red line shows the corresponding change in the normalized conductance *G*/*G*_0_. By plotting the values of *G*/*G*_0_ obtained at the STM tip positions (*x*, *y*, *z*), a 3D volume plot, which shows the change in conductance with the conformational change of the molecular junction, can be obtained (See supplementary information for the comparison with the case of measurement without bonding).

Figure 1e and f show the *G*-*z* curves obtained for one cycle of back and forth motion in the *z*-direction before and after forming a molecular junction. The red and black curves were obtained while the STM tip was retracted from and made to approach the Au(111) surface. When a Au-BDA-Au single-molecule junction was formed, a characteristic *G*-*z* curve with hysteresis, which reflects the change in the conductance of the molecular junction due to the conformational change, was obtained, as shown in Fig. 1f, in contrast to the general exponential decay observed without the BDA molecule shown in Fig. 1e.

### Experimental Results

**Figure 2:**
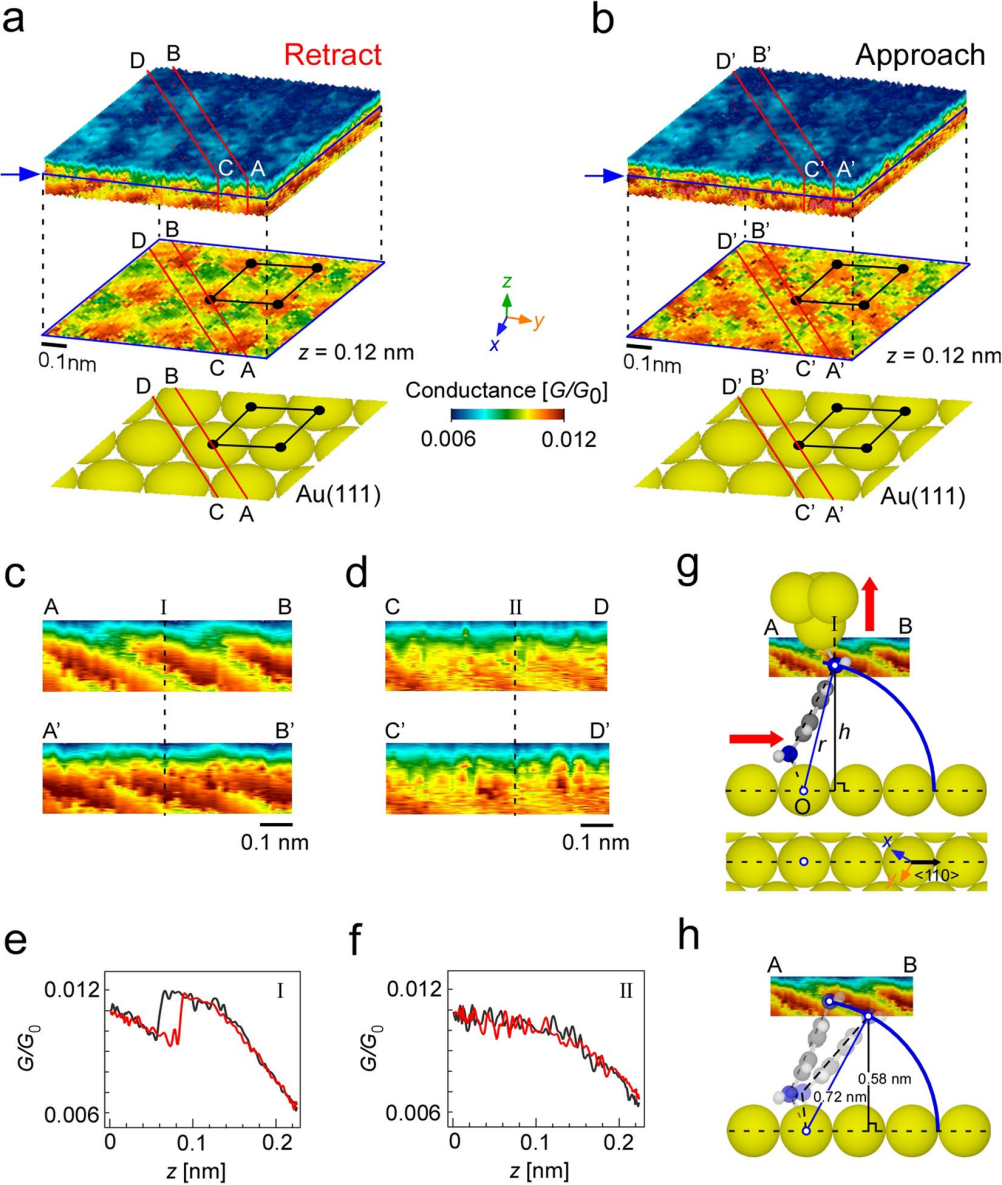
Experimental data. (**a**,**b**) 3D volume plots of conductance obtained by the 3D dynamic probe method shown in Fig. 1 while the STM tip was retracted and made to approach the Au surface, respectively. The *x*-*y* cross sections corresponding to the blue arrows in the volume plot (for *z* = 0.12 nm) and schematic structures of the Au(111) surface are shown together. (**c**,**d**) Cross sections of the volume plots along A-B (A’-B’) and C-D (C’-D’) shown in (**a**,**b**), respectively. (**e**,**f**) *G*-*z* curves along I in ***c*** and II in ***d***, respectively. Red and black lines show the curves obtained when the STM tip was retracted and made to approach the Au surface, respectively. See supplementary information for more details. (**g**) Schematic illustration of the junction structure along with the cross section shown in ***c***. *r* and *h* indicate the N-Au distance and the height of the N atom in the amine on the STM tip side from the center of the Au atom on the substrate, respectively. (**h**) Conformation used to determine *h* (=0.58 nm).

Figure 2a and b show volume plots in the form of conductance maps as a function of the STM tip apex position (*x*, *y*, *z*), which consist of the *G*/*G*_0_ data obtained while the STM tip was retracted from and made to approach the Au surface, respectively (*x*, *y*, *z*: 51 × 51 × 200 points). The *x*-*y* cross sections corresponding to the frames indicated by the blue arrows (*z* = 0.12 nm) and schematics of the Au(111) surface structure are shown together. The high-conductance regions in the *x*-*y* cross sections have the same periodicity as the Au(111) atomic structure. Therefore, it is considered that the substrate-side amine moved reproducibly on the Au(111) surface during measurement, which reflects the change in conductance with the position of the amine on the Au(111) surface, as was observed in our previous study for BDT and BPY^21^. From the high reproducibility of the 3D patterns, the bond structure at the STM tip apex is considered to have been stable during the measurement.

**Figure 3:**
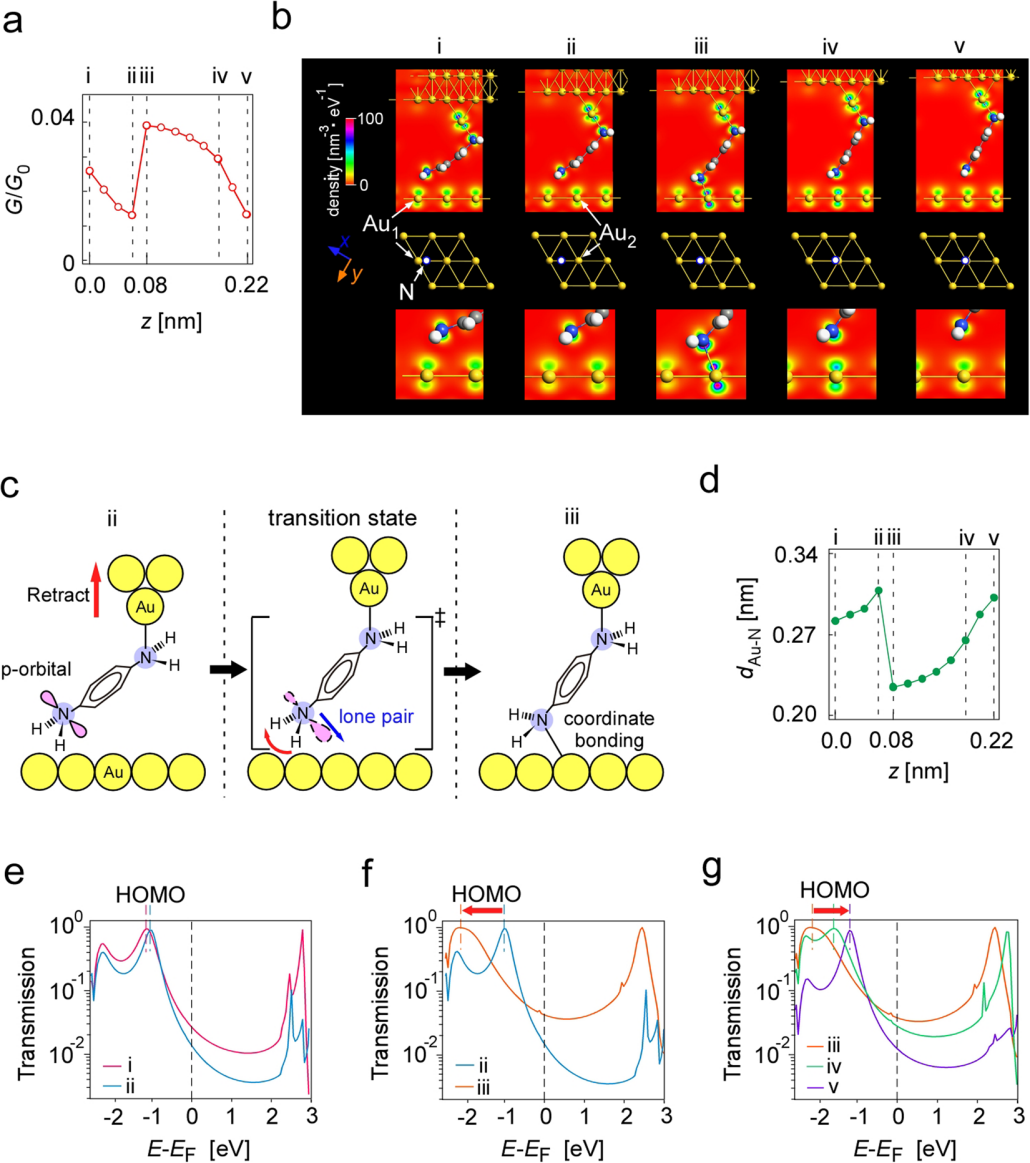
Results of simulation for the molecular movement along a Au atom array. (**a**) *G*-*z* curve obtained by simulation. (**b)** Conformational change in junction and local density of states (LDOS) at i to v in (**a**), viewed for the plane perpendicular to the substrate, which includes the two N atoms in amine. The position of the N atom in the amine on the lower side in each conformation is shown in the top view of the Au substrate structure. (**c)** Model for the conformational change from ii to iii, which changes via a transition state to form a N-Au bond at (iii). (**d)** Distance between N in amine and Au atoms on substrate to which transmission pathway is formed. **(e**,**f**,**g**) Transmissions obtained by simulations for i and ii, ii and iii, and iii, iv and v shown in (**a** and **b)**. The position of the HOMO is indicated by dashed lines and its shift is shown by red arrows.

Figure 2c and d respectively show cross sections of the volume plots along A-B (A’-B’) and C-D (C’-D’) in Fig. 2a,b, which are along a Au atom array and along a line between two Au atom arrays, as indicated by the red lines drawn in the schematics of the Au surface structures shown at the bottom of Fig. 2a and b. Figure 2e and f show the *G*-*z* curves along I in Fig. 2c and II in Fig. 2d, respectively. Red and black lines show the curves obtained when the STM tip was retracted and made to approach the Au surface, respectively. When the amine moved along a Au atom array, a characteristic change in conductance was observed with hysteresis as shown in Fig. 2e, in contrast to the case shown in Fig. 2f, where the amine moved along a line between Au atom arrays. The rapid change in conductance shown in Fig. 2e is considered to be caused by a site-dependence of conductance, and the observed hysteresis is originated from whether the bond is formed or broken, as will be discussed in Fig. 3 (ii to iii) and (iii to ii), respectively. The gradual change in conductance is attributed to the change in transmission due to the conformational change in the junction.

### Simulations

#### Simulation Scheme

To understand this in detail, we carried out simple simulations for these two cases and compared the results with the experimental data. We first consider the case shown in Fig. 2c and e, in which the bright region reproducibly formed arcs. On the basis of the analysis reported in a previous paper^21^, the conformation of the junction can be determined experimentally. The distance *r* between the upper side N and the center of Au on the substrate O shown in Fig. 2g were obtained by optimizing the Au-BDA-Au structure, as 0.72 nm, respectively. Arc with the radius of *r* (=0.72 nm) was drawn with its center located at the center of the Au atom on the substrate, O, as shown in Fig. 2h. The height of the N atom in the amine bonded to the Au atom on the STM tip apex from the center of the Au atom on the substrate, *h*, was estimated to be 0.58 nm (Fig. 2h) in this case, which was used as the initial point in the simulations. Under these conditions, the molecular structure was optimized to prepare its initial state, and the changes in the conformation of junction and its transmission were calculated while the STM tip was retracted in steps of 0.02 nm up to 0.22 nm (11 steps) with an applied bias voltage of 10 mV (see Methods for more details).

#### Results of Simulations

Figure 3a shows the *G*-*z* curve obtained by simulation, which closely reproduced the experimental observation that a rapid increase in conductance occurred when the tip was retracted as shown in Fig. 2c. Figure 3b shows the molecular conformations and the cut planes of local density of states (LDOS) at the five points (i to v) indicated in Fig. 3a, which are perpendicular to the substrate and include the two N atoms. The position of the N atom in the amine on the lower side in each step is superimposed on the top view of the Au substrate shown in the bottom of Fig. 3b. Figure 3c shows the model for the conformational change from ii to iii, which changes via a transition state to form a N-Au bond at (iii). Figure 3d shows the change in *d*_Au-N_ between the N in the amine and the Au atoms on the substrate to which the transmission pathways are formed (Au_1_ and Au_2_ in Fig. 3b). Figure 3e–g show the change in transmission corresponding to the conformational changes form i to ii, ii to iii and iii to v shown in Fig. 3b, respectively, which are explained as follows:

1. i to ii: The p-orbital of N in the lower-electrode (substrate)-side amine weakly interacted the Au atom (Au_1_) and two H atoms faced the Au(111) surface, as shown in Fig. 3c (ii). *d*_Au-N_ increased with increasing distance between the two electrodes, weakening the interaction. Therefore, as shown in Fig. 3e, the highest occupied molecular orbital (HOMO) peak shifted slightly (from −1.12 to −1.02 eV) and its width decreased, resulting in a decrease in the transmission around the Fermi level.
2. ii to iii: As shown in Fig. 3c, the p-orbital in amine became a lone-pair in the transition state as a result of the decrease in *d*_Au-N_ (Fig. 3d) and formed a coordinate bond with a Au atom (Au_2_) on the substrate via the transition state shown in Fig. 3c. The HOMO peak shifted to the lower side owing to the bond formation, and its width increased (Fig. 3f), causing a significant increase in the transmission around the Fermi level.
3. iii to v- The N-Au bond was gradually weakened and *d*_Au-N_ increased as shown in Fig. 3d. As shown in Fig. 3g, the HOMO peak shifted toward the initial position (−1.16 eV) and its width decreased again, reducing the conductance.

As has been shown, the bonding state and molecular conformation of the junction change with the conformational relationship between the N lone-pair in the amine and the Au atom at the substrate.

**Figure 4:**
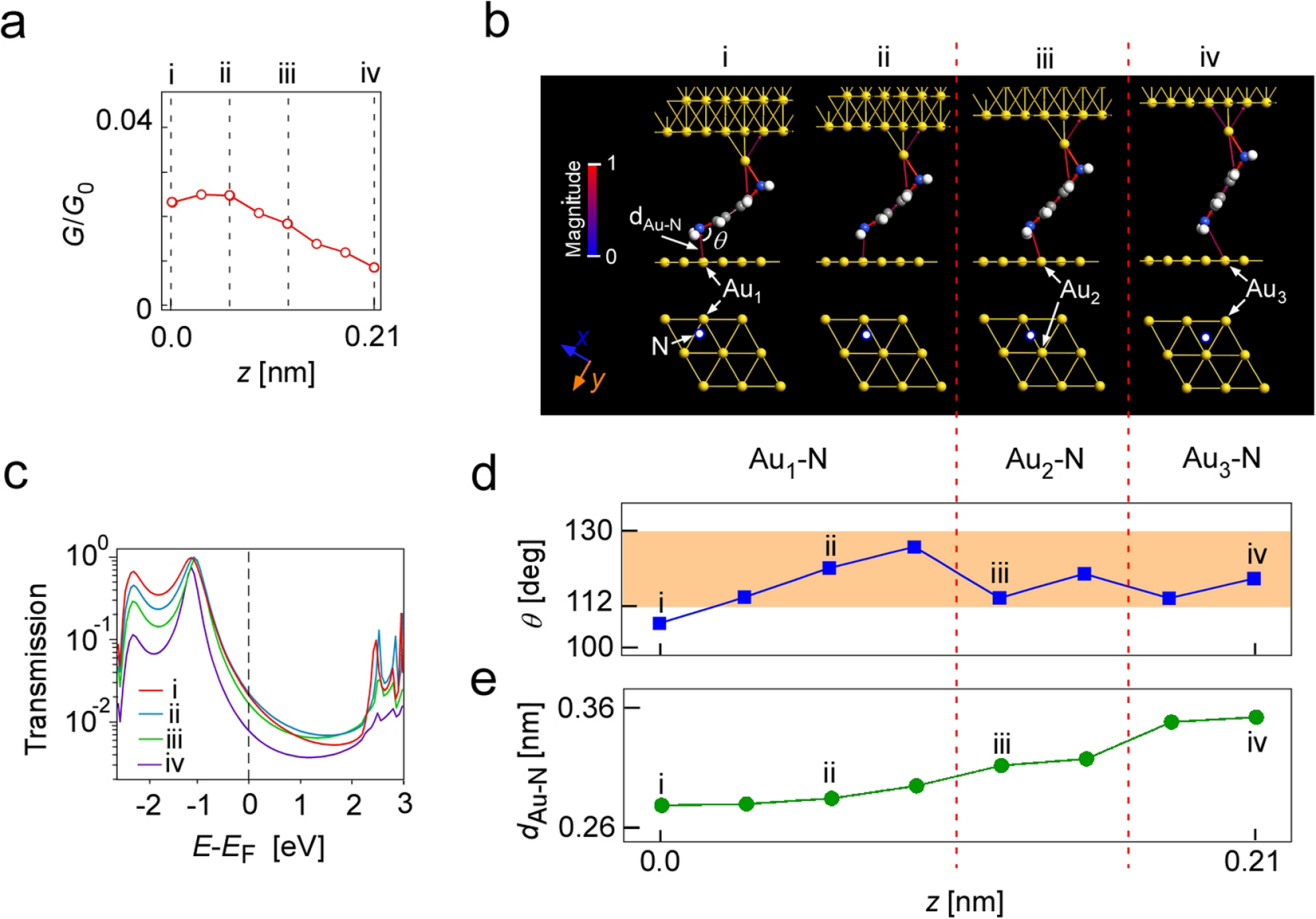
Results of simulation for the molecular movement along a line between two Au atom arrays. (**a)** *G*-*z* curve obtained by simulation. (**b)** Conformations with transmission pathway for i to iv in (**a)**. The position of the N atom in the amine on the lower side is shown in the top view of the Au substrate structure. (**c**) Transmissions obtained for i to iv shown in (**a** and **b**). (**d**) Angle of Au-N-C as a function of the retraction distance, *z*. (**e**) Distance between nitrogen and Au at substrate as a function of *z*.

Next, we carried out simulations for the case in which the amine moved along lines between two Au atom arrays (C-D and C’-D’ in Fig. 2a and b). The initial conformation for this simulation was prepared by optimizing the structure shown in Fig. 2h at a state between two Au arrays. The distance between the two electrodes was increased in steps of 0.03 nm. Figure 4a shows the *G*-*z* curve obtained by this simulation. The conductance changed smoothly and the experimentally observed *G*-*z* curve shown in Fig. 2f was well reproduced.

Figure 4b shows the change in molecular conformation and the transmission pathway corresponding to points i to iv shown in Fig. 4a. The position of the N atom in the amine on the lower side in each step is superimposed on the top view of the Au substrate shown in the bottom of Fig. 4b. Figure 4c shows the transmissions obtained for points i to iv in Fig. 4a. Figure 4d and e show the changes in *θ* and *d*_Au-N_ as the Au atom to which the N atom bonds changes from Au_1_ to Au_3_ (shown in Fig. 4b) as *z* changes. The switching of the Au atom is considered to occur when *θ* became outside the range of 112–130°, which is the range of angles for a stable Au-N-C structure^9^.

In contrast to the case shown in Fig. 3, the HOMO peak position and peak width did not show large changes, which is considered to be due to the absence of coupling of the N lone-pair with the Au atom at the substrate. Taking account of the change in the transmission shown in Fig. 4c and that in *d*_Au-N_ shown in Fig. 4d, the decrease in *G* for *z* > 0.06 nm shown in Fig. 4a is considered to be caused by the decrease in transmission due to the weakening of the interaction resulting from the increase in *d*_Au-N_. The small change in *G* for *z* < 0.06 nm is due to the small change in *d*_Au-N_.

## Conclusions

We have applied our previously developed 3D dynamic probe method to analyze the conductance in a Au-(1,4-benzenediamine)-Au single-molecule junction, which is considered to be the simplest example of single-molecule junction, to measure small variations in its conductance. The effect of the nitrogen (N) lone-pair in the amine group on the conductance, which is difficult to clarify by the analysis by the break junction (BJ) methods, has been revealed for the first time. The bonding state and molecular conformation of the junction changed with the conformational relationship between the N lone-pair in the amine and the Au atom at the substrate. The change in conductance while the molecular conformation was controlled was clearly demonstrated, which showed good agreement with the results of simulations, taking account of the effect of the N lone-pair in an amine bonding with a Au electrode.

## Methods

### Sample and Tip Preparation

A clean flat Au(111) surface was prepared by evaporating Au of 100 nm thickness on a mica substrate, that had been subjected to Ar sputtering (5 min, 1 μA/${{\rm{cm}}}^{2}$) and annealing (30 min, 700 K) for 3~5 cycles. Then the Au(111) surface was exposed to 1,4-benzenediamine (BDA) molecules introduced through a variable leak valve (10 s, partial pressure: 1.0 × ${10}^{-6}$ Pa). The STM tip was formed by cutting a Au wire (0.3 mm diameter).

### Measurement Scheme

All measurements were carried out in vacuum (<5.0 × ${10}^{-8}$ Pa) using an Omicron low temperature STM. By cooling the STM unit with liquid nitrogen for over 1 week, the temperature in the STM chamber was maintained at 83 K and the thermal drift was reduced. After observing the Au(111) surface (*V*s = 0.2 V, *I* = 0.2 nA), the STM tip was moved to above the target molecule. Then the feedback was turned off and the bias voltage was set to 10 mV because the bond was unstable at high bias voltages. The STM tip was moved back and forth in the *z*-direction in accordance with a sinusoidal function (*z*_p-p_ = 0.224 nm), and a two-dimensional scan was carried out step by step after every cycle of *z*-modulation while measuring the current between the tip and substrate. The conductance was measured at each point of the STM tip (*x*, *y*, *z*: 51 × 51 × 200 points in the case of Fig. 1). Before forming the single-molecule junction, an exponential *G*-*z* curve was observed (Fig. 1e). The STM tip was manually made to approach the molecule while observing the change in *G*. When the tip came in contact with the molecule, a rapid increase in conductance was observed and the *G*-*z* curve became nonexponential as shown in Fig. 1f. After forming the single-molecule junction, the tip was retracted until a reproducible *G*-*z* curve was obtained. After observing the stable signal, we started our measurement of the 3D conductance to obtain volume plots as shown in Fig. 2a and b. The effect of the sample gradient was removed by linear correction.

### Simulations

We used Atomistix ToolKit software (version 12. 8.2, Quantum Wise A/S) with density functional theory (DFT) within the generalized gradient approximation (GGA) using the Perdew-Burke-Ernzerhof (PBE) exchange-correlation function^23^ to simulate the optimized structure of the junction and the variation in its conductance while the STM tip was retracted. A double-zeta basis set was used for all atomic species, and the cutoff energy was set to 100 Ry. The sampling for Brillouin zone integration was performed at 3 × 3 × 400 k-points. The conductance was calculated by the nonequilibrium Green’s functional theory (NEGF) method^24,25^. A bias voltage of 10 mV was applied to the electrodes, assumed to comprise three layers of Au(111).

Figure 3b (i) shows the initial conformation. The electrodes consist of three layers of a Au(111) surface. A Au atom (adatom) was added to a hollow site in the Au(111) surface on the upper electrode as the STM tip apex. The height of the upper-electrode-side N atom from the Au(111) surface was set to 0.58 nm, and the N-N axis was placed in the <110> direction. The initial positions of the lower-electrode-side amine on the Au(111) surface in Figs 3b and 4b were selected to reflect the experimental conditions, i.e., the motion of the amine along the Au array and along a line between two Au arrays, respectively.

After the structural optimization and the calculation of the conductance, the distance between the two electrodes was changed in steps of 0.02 nm up to 0.22 nm in Fig. 3 (0.03 nm up to 021 nm in the case of Fig. 4). The upper-electrode-side N atom was fixed during the whole processes to reflect the stable junction structure at the STM tip apex because highly reproducible conductance patterns were obtained in measurements.

Since more detailed analysis is necessary for the calculations while the STM tip is approached, simulations were carried out for the case of STM tip retraction.

## Electronic supplementary material

Supplementary Information
