# Supplementary material for: The effect of nitrogen lone-pair interaction on the conduction in a single-molecule junction with amine-Au bonding
Source: Sci Rep. 2018 Mar 27;8:5222. doi: 10.1038/s41598-018-22893-7 (PMC5869721; doi:10.1038/s41598-018-22893-7)
Supplement: Supplementary file 1 — Supplementary Information [file 41598_2018_22893_MOESM1_ESM.docx]

**Supplementary information**

**The effect of nitrogen lone-pair interaction on the conduction in a single-molecule junction with amine-Au bonding**

Yoshihiro Sugita, Atsushi Taninaka, Shoji Yoshida, Osamu Takeuchi, and Hidemi Shigekawa

Faculty of Pure and Applied Sciences, University of Tsukuba, Tsukuba 305-8571, Japan


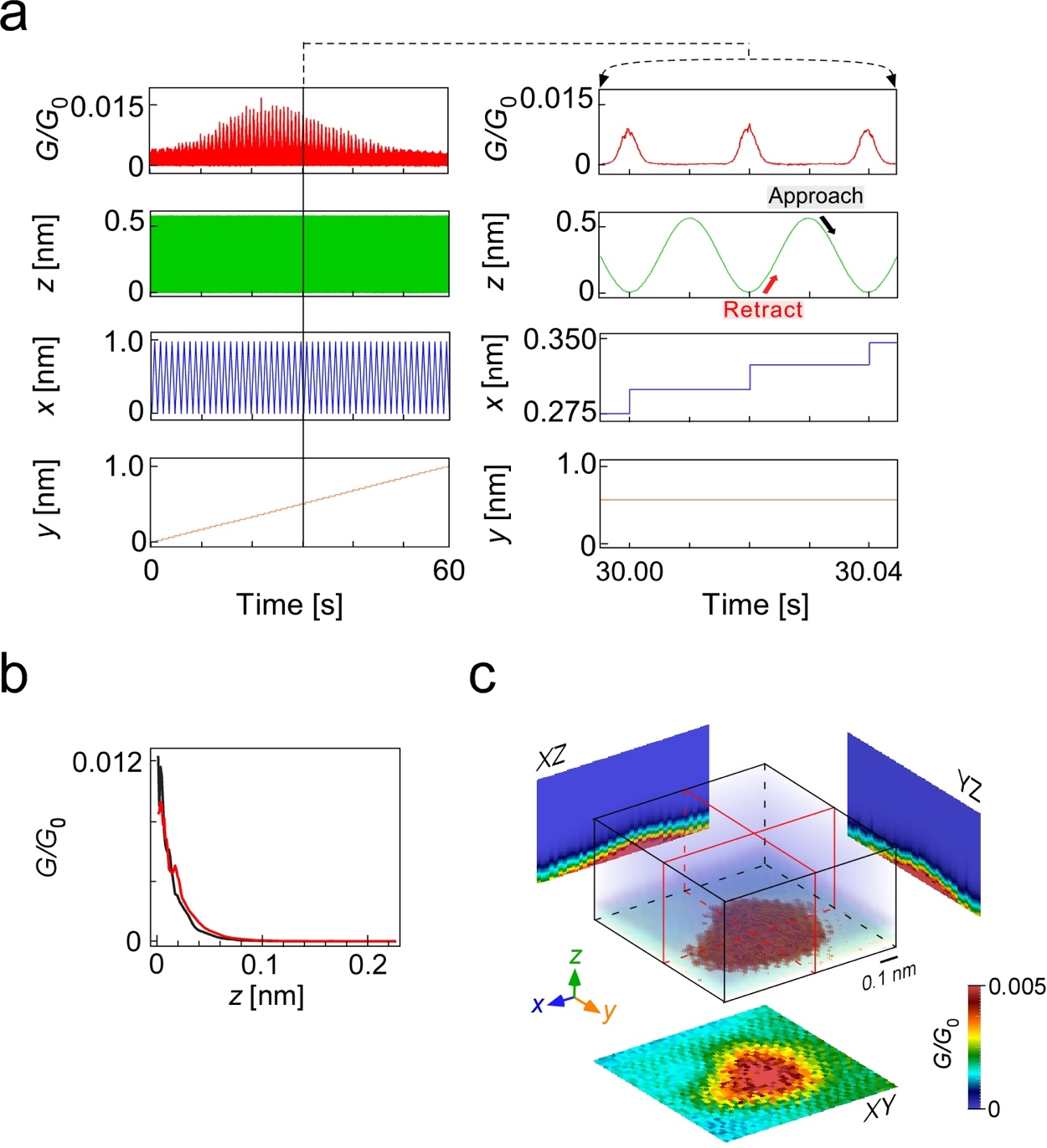


Fig. S1 **Measurement scheme and example of data for BDA/Au(111).** ***a*** Measurement scheme and an example of a signal. A high-conductance signal was obtained above the molecule. ***b*** *G*-*z* curves showing an exponential characteristic. ***c*** Volume plot of the 3D dynamic probe data obtained for a BDA/Au(111) surface before forming a junction (data for STM tip retraction). The cross sections correspond to the frames indicated by the red lines in the volume plot.


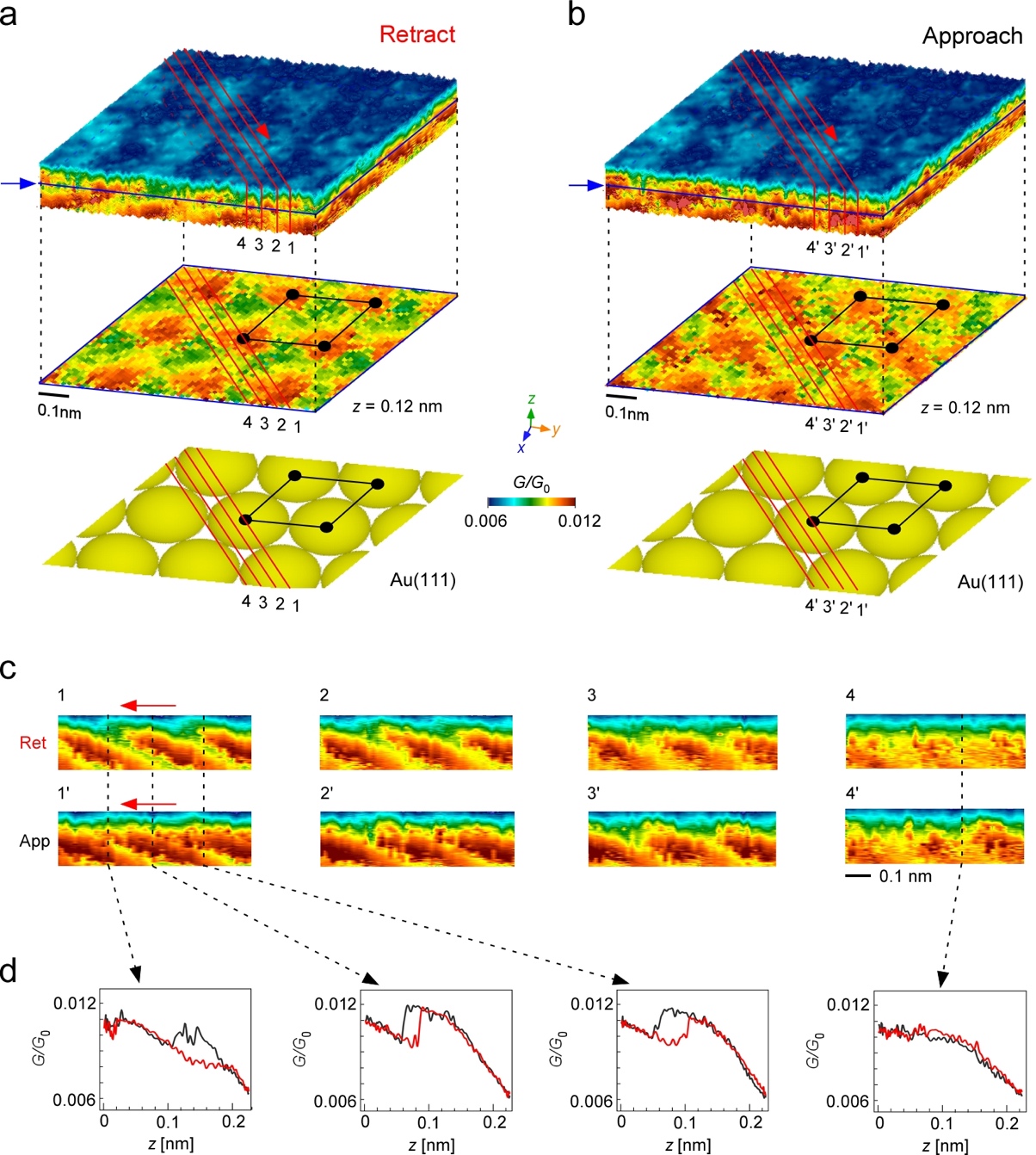


Fig. S2 **Experimental data**. (***a***, ***b***) 3D volume plots of conductance obtained by the 3D dynamic probe method shown in Fig. 1 while the STM tip was retracted and made to approach the Au surface, respectively. The *x*-*y* cross sections corresponding to the blue arrows in the volume plot (for *z* = 0.12 nm) and schematic structures of the Au(111) surface are shown together. ***c*** Cross sections of the volume plots along 1 to 4 and 1’ to 4’ shown in ***a*** (***b***), respectively. The red arrows drawn in 1 and 1’ show the direction of the cross-sections as indicated by the arrows drawn in the 3D plots in ***a*** and ***b***. ***d*** *G*-*z* curves along the dotted lines in ***c***. Red and black lines show the curves obtained when the STM tip was retracted and made to approach the Au surface, respectively. The observed change in the three *G*-*z* curves obtained for the cross-section 1 is explained by the positional difference (shift along AB) for the back and force measurement. About the change from 1 to 4 in ***c*** and ***d***, the image of arc becomes dull and the rapid change in the *G*-*z* curve was reduced, which is considered to be due to the change in the conformational relationship between the N lone-pair in the amine and the Au atom at the substrate. For further understanding, detailed simulations of the *G*-*z* curves for the two lines between AB and CD are necessary, which we remain for future work.
